# Genotype-phenotypic correlation in a Chinese patient with isolated lissencephaly sequence caused by 17p13.3p13.2 chromosomal microdeletion: a 6-year follow-up study

**DOI:** 10.3389/fgene.2026.1866451

**Published:** 2026-09-09

**Authors:** Jiao Tong, Xu Chen, Tao Wang, Shan Ma, Yali Zhao, Dongdong Shi, Xin Wang, Dongmei Yan

**Affiliations:** Lianyungang Maternal and Child Health Hospital Affiliated to Kangda College of Nanjing Medical University, Lianyungang, China

**Keywords:** 17p13.3p13.2 microdeletion, genotype-phenotype correlation, isolated lissencephaly sequence, longitudinal follow-up, PAFAH1B1

## Abstract

**Background:**

Isolated lissencephaly sequence (ILS) is a severe neurodevelopmental disorder associated with 17p13.3 microdeletion. This 6-year longitudinal study aimed to systematically characterize physical and neurodevelopmental trajectories of a Chinese ILS patient and offer evidence for early diagnosis and clinical intervention.

**Methods:**

From April 2021 to April 2026, a child with severe developmental delay and his family members (parents and elder brother) were recruited in Lianyungang, eastern China. Trio whole-exome sequencing (trio-WES) and copy number variation sequencing (CNV-seq) were used to identify the pathogenic variant. Serial physical growth and neurodevelopmental assessments were conducted during the 6-year longitudinal follow-up. Bioinformatics analysis was used to explore potential molecular pathogenic mechanisms.

**Results:**

A *de novo* 2.06 Mb heterozygous deletion at 17p13.3p13.2 (chr17:1707883_3765621del, GRCh37) was identified in the proband, which included *PAFAH1B1* but spared *YWHAE* and *CRK*. Longitudinal data showed a progressive decline in both height and weight. Height Z score decreased from −0.37 at 2 months to −1.92 at 57 months. Weight Z score decreased from −1.25 at 2 months to −1.79 at 57 months. Gesell developmental quotients (DQ) showed significant progressive declines in five domains with a decelerating trajectory: adaptive behavior (B_2_ = 0.019, 95% *CI*: 0.002–0.036, *R*_*Q*_^*2*^ *=* 0.978), gross motor (B_2_ = 0.013, 95% *CI*: -0.004 – 0.030, *R*_*Q*_^*2*^ *=* 0.944), fine motor (B_2_ = 0.017, 95% *CI*: -0.012 – 0.047, *R*_*Q*_^*2*^ *=* 0.870), language (B_2_ = 0.021, 95% *CI*: -0.006 – 0.049, *R*_*Q*_^*2*^ *=* 0.905), personal social behavior (B_2_ = 0.020, 95% *CI*: -0.014 - 0.054, *R*_*Q*_^*2*^ *=* 0.847). Bioinformatics analysis confirmed that haploinsufficiency of the *PAFAH1B1* gene was the primary pathogenic cause. Furthermore, genes in the deleted region were significantly enriched in olfactory perception and calcium ion transmembrane transport pathways.

**Conclusion:**

This study reports a 6-year longitudinal follow-up of ILS in a Chinese patient. *PAFAH1B1* haploinsufficiency causes the core lissencephaly phenotype, while co-deletion of olfactory and calcium-regulatory genes may exert synergistic effects. These findings expand the phenotypic spectrum of ILS in Chinese populations and provide insights for mechanistic studies and genetic counseling.

## Introduction

Chromosome 17p13.3 represents a genomically unstable region implicated in a wide spectrum of neurodevelopmental disorders. This region harbors a high density of low-copy repeats and is thus widely recognized as a “recombination hotspot” (Ji et al., 2025; Blazejewski et al., 2018). Heterozygous deletions of variable-sized fragments within this region can cause a continuum of phenotypic alterations, leading to neuronal migration disorders and various forms of brain malformations, including lissencephaly (Wade et al., 2024; Dobyns et al., 1993). Lissencephaly, literally meaning “smooth brain,” is characterized by a smooth or nearly smooth cerebral cortex accompanied by hypoplastic gyri and sulci. Its phenotypic spectrum ranges from complete agyria to subcortical band heterotopia (SBH) (Kato and Dobyns, 2003). Moreover, the malformation spectrum associated with 17p13.3 microdeletions extends from isolated lissencephaly sequence (ILS; OMIM 607432) to Miller-Dieker syndrome (MDS; OMIM 247200) (Ji et al., 2025; Romano et al., 2020; Mahendran and Brown, 2025). Although lissencephaly is a core feature in both conditions, ILS and MDS are recognized as distinct diseases in terms of both genotype and phenotype. MDS typically manifests with classical lissencephaly and distinctive facial dysmorphisms, corresponding to the most severe phenotypic variant (Mahendran and Brown, 2025). In contrast, patients with ILS lack the characteristic facial dysmorphisms and exhibit a relatively milder degree of lissencephaly (Mahendran and Brown, 2025).

Isolated lissencephaly sequence (ILS) is a rare and severe congenital neurological disorder (Chong et al., 1996). In addition to the core cerebral malformation, affected individuals manifest a spectrum of severe clinical features, including global developmental delay, myoclonic jerks and spasms, intellectual disability, hypotonia, epileptic seizures, motor coordination deficits, and microcephaly (Blazejewski et al., 2018; Leventer et al., 2001; Brock et al., 1993). Sensory abnormalities, cognitive impairment, feeding difficulties, constipation, central visual impairment, and scoliosis are also common clinical manifestations (Brock et al., 1993; Pavone et al., 1993; Dobyns et al., 1992). Typical imaging and pathological findings of lissencephaly include a smooth or nearly smooth cerebral surface with underdeveloped gyri and sulci (Curry et al., 2013a; Leibovitz et al., 2022). Additionally, the cerebral cortex is abnormally thickened and disorganized, with widespread neuronal heterotopia, enlarged and malformed ventricles, and frequent agenesis of the corpus callosum (Curry et al., 2013a; Leibovitz et al., 2022; Lo Nigro et al., 1997).

At the genetic level, the chromosome 17p13.3 region harbors two key genes of interest: *PAFAH1B1* (OMIM 607432) and *YWHAE* (OMIM 605066) (Blazejewski et al., 2018). Deletion or duplication affecting either of these genes can result in abnormal brain development (Blazejewski et al., 2018; Curry et al., 2013b; Denommé-Pichon et al., 2023; Cardoso et al., 2002). Approximately 60% of ILS cases are caused by *PAFAH1B1* haploinsufficiency (Ji et al., 2025). In contrast, MDS involves the concurrent deletion of both *PAFAH1B1* and *YWHAE* (Mahendran and Brown, 2025). Previous studies have indicated that *PAFAH1B1* plays a critical role in early embryonic development, and haploinsufficiency of this gene can lead to classical lissencephaly (Mahendran and Brown, 2025; Bruno et al., 2010; Zhang et al., 2022). Moreover, isolated *PAFAH1B1* deletion is a common cause of classical grade 2–4 lissencephaly (Ji et al., 2025; Lo Nigro et al., 1997). Clinical manifestations include smooth brain, ranging from varying degrees of mixed agyria/pachygyria or solely pachygyria, and no facial dysmorphism (Yingling et al., 2003). When the 17p13.3 deletion extends to a contiguous ∼1.3-Mega base (Mb) pair region that includes both *PAFAH1B1* and *YWHAE*, it leads to the more severe MDS (Brock et al., 1993). In addition, other copy number variations (CNVs), such as 17p13.3 microdeletions and microduplications, resulting from various combinations of *PAFAH1B1* and *YWHAE* (Nagamani et al., 2009; Emrick et al., 2019). Therefore, delineating the clinical phenotypes of patients and understanding the impact of relevant genetic variations on individual growth and development are of great significance for exploring the pathogenic mechanisms of this disorder.

Due to the complex phenotypes, the early screening and diagnosis of 17p13.3 microdeletion face numerous challenges. Influenced by factors such as variant type, differences in gene penetrance, resolution limitations of detection methods, and gestational age, these variants often fail to exhibit detectable or specific imaging features during the fetal period. In recent years, molecular genetic testing technologies have developed rapidly. Techniques such as trio whole-exome sequencing (Trio-WES) and copy number variation sequencing (CNV-Seq) allow effective identification of genome-wide CNVs and precise mapping the variant regions. These approaches have improved the detection efficiency and accuracy of pathogenic genomic variants, providing important technical support for early diagnosis of developmental disorders (Lu et al., 2023; Huang et al., 2026; Zhang et al., 2024). However, to date, most global studies on 17p13.3 microdeletion-related ILS have been concentrated in Europe and the United States, with extremely limited research originating from China (Wang et al., 2023; Lin et al., 2017; Zhang et al., 2020). This significantly hinders the understanding of the natural disease course, developmental trajectory, and prognostic characteristics. Accordingly, studies focusing on 17p13.3 microdeletion and long-term prognosis follow-up in the Chinese population need to be further accumulated and improved.

In this study, Trio-WES combined with CNV-Seq technology was used to conduct genetic testing on a Chinese boy with ILS caused by a 17p13.3p13.2microdeletions and his family members (parents and elder twin brother). Through 6 years of longitudinal follow-up, this study systematically analyzed the genotype-phenotype correlation of this microdeletion and evaluated its long-term impact on the physical growth and neuropsychological development. The analysis further explored the synergistic contribution of olfactory and calcium-related genes within the deleted region. These findings help to further improving the phenotypic characteristics of ILS in the Chinese population, and provide an important theoretical basis for accurate clinical diagnosis and genetic counseling.

## Materials and methods

### Research subject

A male patient with severe global developmental delay and his family (parents and elder twin brother) were recruited from Lianyungang Maternal and Child Health Hospital in April 2021. This study was approved by the Medical Ethics Committee of Lianyungang Maternal and Child Health Hospital (Project No.: XM2022030). Written informed consent was obtained from the legal guardians of the proband prior to participation, in accordance with the principles of the Declaration of Helsinki.

### Collection of clinical data, diagnosis and functional assessments

Relevant clinical data of the proband, his elder brother, and parents were collected, including (Ji et al., 2025): present illness history and family history (Blazejewski et al., 2018); physical examination findings (Wade et al., 2024); auxiliary examination results, including developmental functional assessments, ultrasonographic imaging data and brain MRI. All clinical data were extracted from medical records. The follow-up data was collected by professional clinicians through face-to-face consultation, telephone communication, online interview and other approaches.

Height and weight of the proband were measured according to the Updated Growth Standards for Chinese Children Under 7 Years of Age (WS/T 423–2022) (Zong et al., 2023). Deviations of height and weight from reference population measurements of the same age and sex was expressed as Z scores (Zong et al., 2023; Zhang et al., 2021; Fenton et al., 2024). Given his preterm birth, corrected age was used for growth assessments up to 24 months of age. The developmental quotient (DQ) of the proband was assessed by qualified clinicians using the Gesell Developmental Schedule (GDS) in accordance with the Diagnostic and Statistical Manual of Mental Disorders, 5th Edition (DSM-5) (Association, 2013; Liu et al., 2025). This scale covers five core domains: adaptive behavior, gross motor skills, fine motor skills, language, and personal social behavior (Liu et al., 2025). Multiple studies have confirmed that this schedule has good reliability and validity in Chinese population (Zhang et al., 2024; Tang et al., 2008; Wang et al., 2016; Ding et al., 2015). Notably, the proband received no rehabilitation intervention, and no epileptic symptoms were reported during the follow-up period.

### Statistical analysis

Statistical analysis was performed on the longitudinal follow-up data of Gesell developmental quotient for the proband. The DQ scores of each domain were continuous variables and did not follow a normal distribution. To characterize the dynamic trajectory of DQ changes with age, a linear and nested second-order polynomial regression models was fitted (Flom et al., 2017). *R* ^*2*^ refers to coefficient of determination, indicating goodness of fit. *R*-squared change (*ΔR* ^*2*^) was examined to determine if adding a quadratic component to the data significantly increased the amount of variance in DQ explained by the model. Age in months was used as the independent variable and the DQ of each domain as the dependent variable, to more precisely describe the nonlinear pattern of developmental decline over time. All statistical analyses were two-sided. All statistical analyses were performed using GraphPad Prism 9.0 (GraphPad Software, San Diego, CA, United States) and SPSS 26.0 (IBM Corp., Armonk, NY, United States).

### Genomic DNA extraction

Peripheral venous blood (2 mL) was collected from the proband, his elder brother and parents and placed in EDTA anticoagulation tubes. Genomic DNA (gDNA) was extracted from 500 µL anticoagulated blood using the Blood Genomic DNA Extraction Kit (Tiangen Biotech (Beijing) Co., Ltd.) following the manufacturer’s protocol. The concentration and purity of the extracted DNA were determined using a NanoDrop 2000 ultra-micro spectrophotometer (Thermo Fisher Scientific, United States), and only DNA samples with an A260/A280 ratio of 1.8–2.0 were used for subsequent experiments.

### Trio whole exome sequencing (Trio-WES)

After extracting genomic DNA from the proband, his elder brother, and parents, library construction was performed according to the standard protocol. Isolated gDNA underwent ultrasonic fragmentation with a Covaris system, followed by terminal repair, amplification and cyclization to generate DNA nanoballs. Subsequently, exome sequencing was then performed on the DNBSEQ-T7 sequencing platform (BGI, Shenzhen, China) with an average sequencing depth of 150× and Q30 > 90%. Raw NGS FASTQ reads were mapped against the human reference genome (GRCh37/hg19) with the Burrows-Wheeler Aligner (BWA). After data filtering, clean reads for each sample were used for subsequent bioinformatics analysis. The detailed exome sequencing protocol is described in the previous research (Lappalainen et al., 2019). All genetic variants were annotated and filtered, and classified according to the pathogenicity scoring system of the American College of Medical Genetics and Genomics (ACMG) (Riggs et al., 2020). CNVs identified by Trio-WES were further verified by CNV-Seq.

### CNV-seq validation

Genomic DNA (gDNA) was fragmented and prepared into sequencing libraries using a BGI library construction kit (BGI Tech, Shenzhen, China). Sequencing was performed on the MGISEQ-2000 high-throughput sequencing platform. Raw data were aligned to the human reference genome assembly (GRCh37/hg19) using the BGI OmicsOne bioinformatics platform with the CombVar analysis pipeline. The sample was analyzed for chromosomal aneuploidy and CNVs larger than 100 kb. Identified CNVs from Trio-WES or CNV-seq were filtered and annotated using the Database of Genomic Variants (DGV; http://dgv.tcag.ca). The pathogenicity of each CNV was classified according to the joint guidelines issued by the ACMG and ClinGen (Riggs et al., 2020).

### Bioinformatics analysis

Databases used for variant annotation included gnomAD (https://gnomad.broadinstitute.org/), DGV (https://dgv.tcag.ca/), dbSNP (https://www.ncbi.nih.gov/projects/SNP/), DECIPHER (https://www.deciphergenomics.org/), ClinGen (https://clinicalgenome.org/), OMIM (https://omim.org/), UCSC Genome Browser (https://genome.ucsc.edu/), and ClinVar (https://www.ncbi.nlm.nih.gov/clinvar/). Candidate variants were classified and their pathogenicity was evaluated according to the ACMG guidelines (Riggs et al., 2020).

To characterize gene dosage effects and pathogenicity within the proband’s deleted region, haploinsufficiency prediction (pHaplo) and loss-of-function intolerance (pLI) analyses were first conducted on the protein-coding genes. These analyses were used to evaluate their dosage sensitivity and evolutionary conservation. Meanwhile, the present study performed Gene Ontology (GO) enrichment analysis on protein-coding genes within the 17p13.3p13.2 microdeletion region via R software (version 4.5.3). The analysis included three categories: biological process, cellular component and molecular function. The adjusted *P* value (*P*_*.adj*_) < 0.05 was set as the significance threshold. The significantly enriched GO terms were visualized using a bubble plot.

## Results

### Maternal pregnancy and perinatal conditions

The proband’s parents were not consanguineous. The mother had no high-risk factors such as high fever, rash, or exposure to toxic substances or radiation during pregnancy. She conceived via *in vitro* fertilization and embryo transfer (IVF-ET) for infertility, and two fresh embryos successfully transferred. Standardized tocolytic therapy was administered thereafter and continued beyond 12 weeks of gestation. Prenatal abdominal color Doppler ultrasonography at 50 days of gestation indicated a dichorionic diamniotic twin pregnancy. Gestational diabetes mellitus was diagnosed at 28 weeks of gestation. Blood glucose was managed by exercise and dietary adjustment. However, no regular blood glucose monitoring and no insulin treatment was administered during pregnancy. No abnormal manifestations were found in other family members.

The mother presented with vaginal fluid leakage at 31+2 weeks of gestation without abdominal pain or vaginal bleeding. She was admitted to hospital as an emergency at 31+5 weeks of gestation due to fever and persistent vaginal fluid leakage. Preliminary diagnosis included twin pregnancy (dichorionic diamniotic), premature rupture of membranes (3 days), fever of unknown origin (suspected chorioamnionitis), gestational diabetes mellitus, post IVF-ET, primipara, breech presentation of twins. Cesarean section was performed subsequently. Regular prenatal examinations were unremarkable. No obvious structural abnormalities in Down syndrome screening and fetal systematic color Doppler ultrasound screening. Thyroid function was normal.

### Physical growth and craniofacial characteristics of the proband

The proband was a male, G1P1, the younger of twin, born prematurely at 31+5 weeks of gestation due to premature rupture of membranes in April 2021 with a birth weight of 1.75 kg. The Apgar score was 9 at 1 min (1 point deducted for skin color) to 10 at 5 min. Amniotic fluid was clear. He was admitted to the neonatal department for treatment after delivery. Follow-up at our hospital began in July 2021. At the initial follow-up, the proband had an actual age of 2 months and 23 days (26 days of corrected age). He was alert and in a good mental state. No jaundice was observed in the skin and sclera; the anterior fontanel measured 1.2 cm × 1.5 cm. In addition, the neck was supple, respiration was stable, bilateral breath sounds were clear, and heart rhythm was regular. No obvious abnormalities were found on abdominal examination, and limb muscle tone was normal.

**FIGURE 1:**
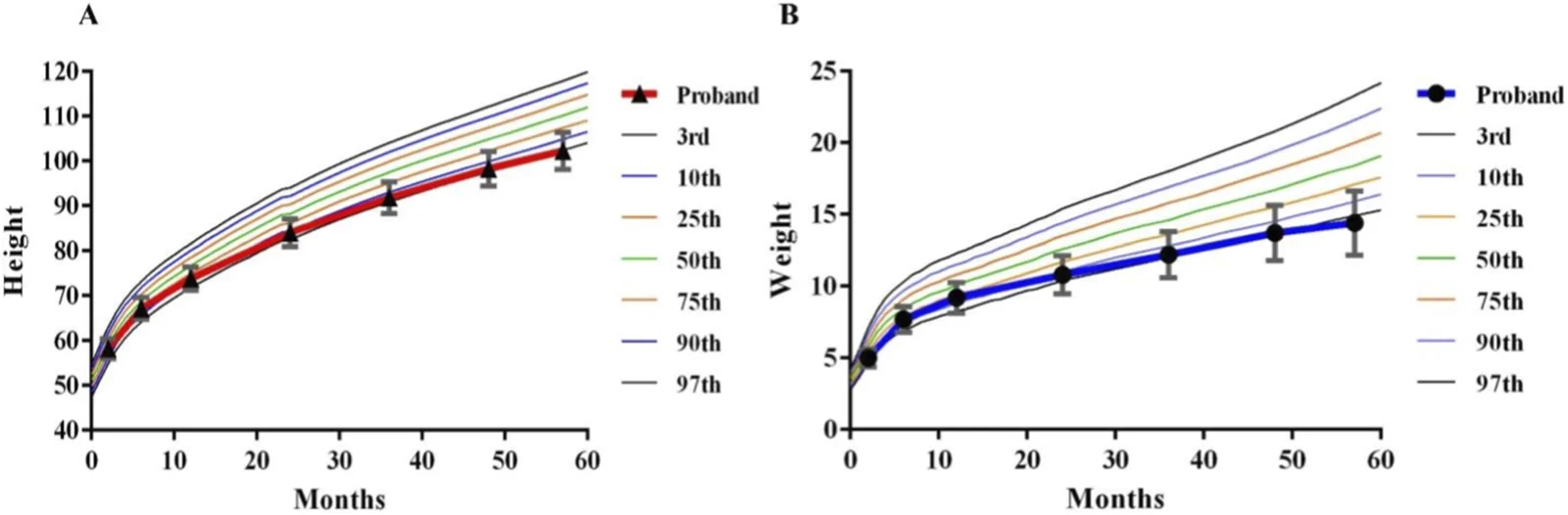
Height and weight growth curves of the proband aged 2–57 months. The sex-specific reference curves were presented using percentiles (3rd, 10th, 25th, 50th, 75th, 90th, 97th), based on the Growth Standards for Chinese Children Under 7 Years of Age (WS/T 423–2022) (Zong et al., 2023). **(A)** Height growth curve: The proband’s linear growth trajectory was marked by a red line with black triangles; gray error bars indicated the SD of the reference population. The height Z score decreased from −0.37 at 2 months to −1.92 at 57 months (Table 1, Supplementary file 1). **(B)** Weight growth curve: The proband’s weight gain was shown as a blue line with black circles, with gray error bars denoting the SD of the reference population. The weight Z score decreased from −1.25 at 2 months to −1.79 at 57 months (Table 2, Supplementary file 1). The horizontal axis indicated age in months, while the vertical axis represented height (cm) in **(A)** and weight (kg) in **(B)**.

In accordance with the Updated Growth Standards for Chinese Children Under 7 Years of Age (WS/T 423–2022) (Zong et al., 2023), height and weight data were collected at corrected ages of 2, 6, 12, and 24 months, and chronological ages of 36, 48, and 57 months. Height-for-age and weight-for-age curves were constructed (Figures 1A,B). The proband’s linear growth trajectory was shown as a red line with black triangles, while the gray error bars represented the standard deviation (SD) of the reference population. According to the growth curves, the proband’s height increased progressively with age and followed the general growth pattern similar to healthy children. However, his height Z score decreased from −0.37 at 2 months to −1.92 at 57 months (Table 1, Supplementary file 1). The proband’s weight gain was presented as a blue line with black circles, with gray error bars indicating the SD of the reference population. Similarly, weight Z score decreased from −1.25 at 2 months to −1.79 at 57 months (Table 2, Supplementary file 1). At 57 months of age, the proband’s height and weight Z scores were below the reference range, indicating impaired linear growth and weight gain.

**TABLE 1:** Height, standard deviation, and Z score of the proband at different ages.

Age	Height (cm)	SD	Z
2	58.2	2.15	−0.37
6	67.2	2.39	−0.63
12	73.8	2.63	−1.10
24	84.0	3.09	−1.36
36	91.8	3.51	−1.62
48	98.3	3.88	−1.70
57	102.3	4.12	−1.92

SD: standard deviation, cm: centimeter, Z = $\frac{X‐\text{mean}}{\text{SD}}$ .

**TABLE 2:** Weight, standard deviation, and Z score of the proband at different ages.

Age	Weight (kg)	SD	Z
2	5.0	0.64	−1.25
6	7.7	0.90	−0.78
12	9.2	1.06	−0.85
24	10.8	1.33	−1.35
36	12.2	1.60	−1.50
48	13.7	1.92	−1.56
57	14.4	2.23	−1.79

SD: standard deviation, kg: kilogram, Z = $\frac{X‐\text{mean}}{\text{SD}}$ .

**FIGURE 2:**
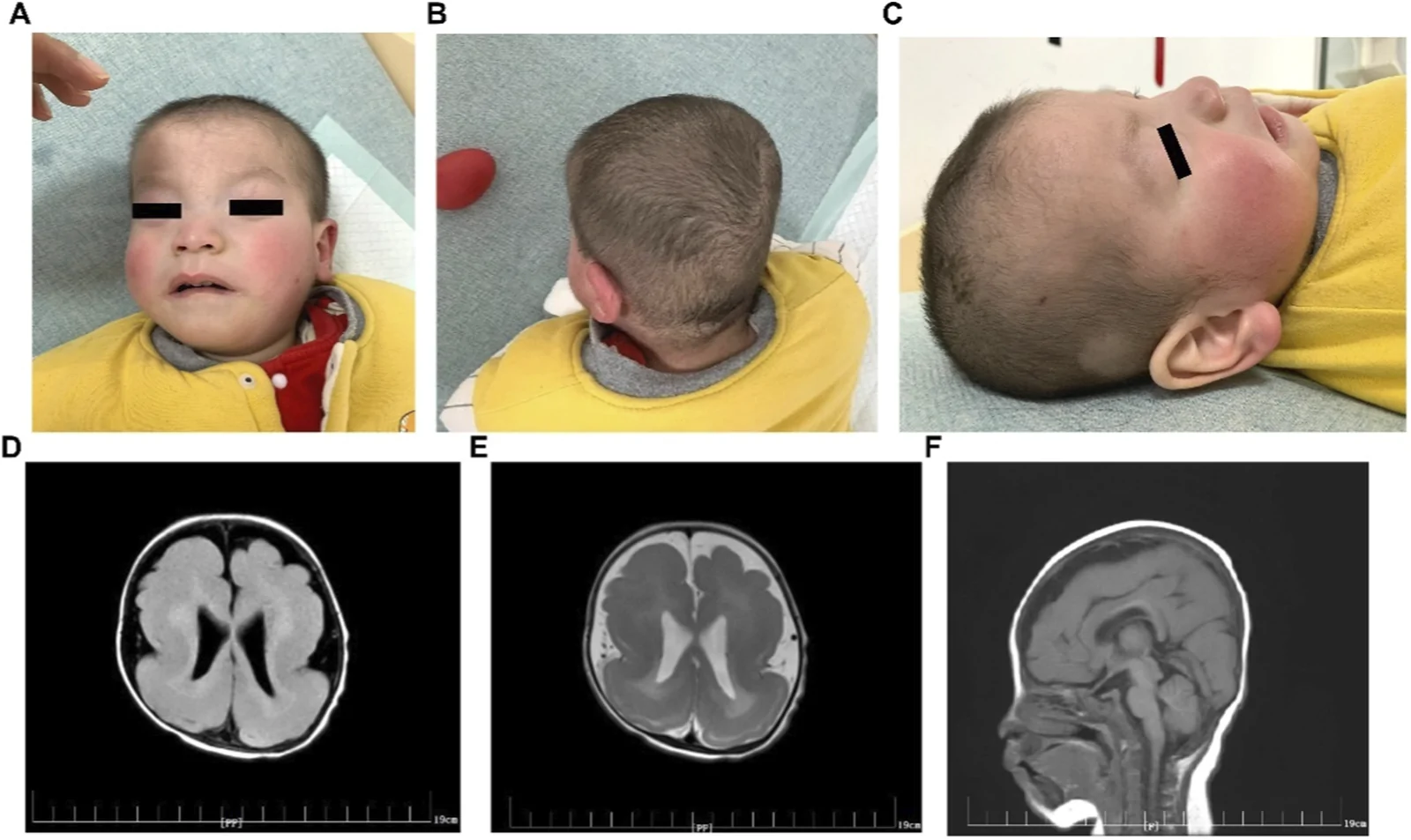
Craniofacial features and brain MRI imaging findings of the proband. **(A–C)** Photographs of the proband showing typical facial features of ILS, including microcephaly, prominent forehead, midface hypoplasia, and characteristic dysmorphic facies. **(D–F)** Cranial MRI scans showing abnormal neuronal migration. Axial views demonstrate widened gyri, shallow sulci, and a simplified cerebral cortex. Sagittal view reveals unclear morphological definition of the rostrum and genu of the corpus callosum, suggestive of hypoplasia or delayed myelination.

The proband exhibited typical facial features of ILS, including microcephaly, plagiocephaly, prominent forehead, midface hypoplasia, hypertelorism and characteristic dysmorphic facies (Figures 2A–C). Cranial magnetic resonance imaging (MRI) revealed broad, smooth gyri and attenuated sulcation with focal cortical thickening in the bilateral frontoparietal lobes, accompanied by a marked reduction in cerebral white matter volume across both cerebral hemispheres. The rostrum, genu, and body of the corpus callosum were indistinct on imaging, and separation of the bodies of the bilateral lateral ventricles was noted (Figures 2D–F). In addition, unclear signal intensity and morphological definition were observed in the rostrum and genu of the corpus callosum, suggesting delayed myelination or congenital hypoplasia in these regions.

### Longitudinal neurodevelopmental follow-up of the proband

**FIGURE 3:**
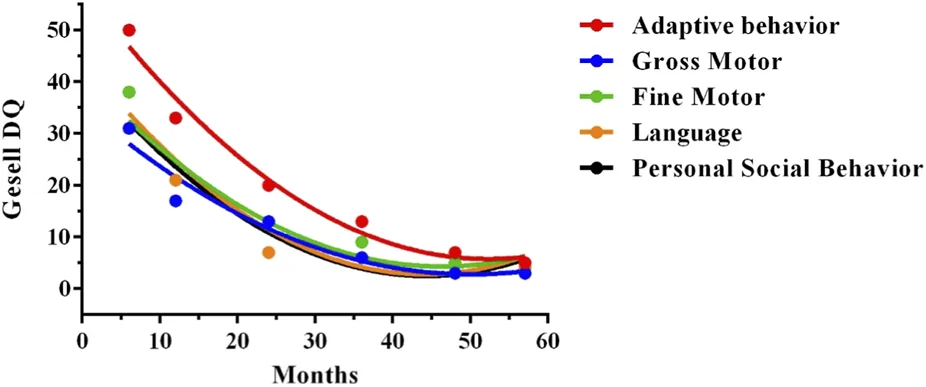
Longitudinal trajectories of Gesell Developmental Quotient (DQ) across five developmental domains in the proband. Scatter points represent raw DQ scores, and solid lines represent second-order polynomial regression fitting curves. All domains exhibit a consistent nonlinear pattern of age-related decline with a decelerating rate, with the most marked trajectory observed in adaptive behavior (Table 3, Supplementary file 1). Adaptive behavior (red), Gross motor (blue), Fine motor (green), Language (orange), Personal Social behavior (black).

Neurodevelopmental assessments were performed by specialist clinicians using the Gesell Developmental Schedule (Liu et al., 2025). Assessments were conducted at corrected ages of 6, 12, and 24 months, and at chronological ages of 36, 48, and 57 months. To characterize age-dependent developmental trajectories across five functional domains, second-order polynomial regression models was fitted (Figure 3). The developmental quotient (DQ) was set as the dependent variable and age in months was set as the independent variable. This analysis aimed to analyze the nonlinear trend of the developmental quotient changing with age in different fields. All regression models exhibited acceptable goodness-of-fit statistics (Table 3, supplementary file 1). Table 3 reports the regression coefficients for the full quadratic model and summarizes the linear *R* ^*2*^, quadratic *R* ^*2*^ and *ΔR* ^*2*^ after including the curvilinear component. *R*-squared change (*ΔR* ^*2*^) was examined to determine if adding a quadratic component to the data significantly increased the amount of variance in DQ explained by the model. Specifically, adaptive behavior (B_2_ = 0.019, 95% *CI*: 0.002–0.036, *R*_*Q*_^*2*^ *=* 0.978), gross motor (B_2_ = 0.013, 95% *CI*: -0.004 – 0.030, *R*_*Q*_^*2*^ *=* 0.944), fine motor (B_2_ = 0.017, 95% *CI*: -0.012 – 0.047, *R*_*Q*_^*2*^ *=* 0.870), language (B_2_ = 0.021, 95% *CI*: -0.006 – 0.049, *R*_*Q*_^*2*^ *=* 0.905), personal social behavior (B_2_ = 0.020, 95% *CI*: -0.014 - 0.054, *R*_*Q*_^*2*^ *=* 0.847). All neurodevelopmental domains showed an age-related reduction in DQ scores, and the decline slows progressively over time. The adaptive behavior domain displayed the most marked alteration.

**TABLE 3:** Quadratic polynomial regression of Gesell Developmental Schedule DQ scores against months in the proband.

Domain	Linear B_1_ [95% CI]	Quadratic B_2_ [95% CI]	R_L_^2^	R_Q_^2^	ΔR^2^
Adaptive behavior	−2.009 [-3.104; −0.915]	0.019 [0.002; 0.036]	0.884	0.978	0.094
Gross motor	−1.302 [-2.390; −0.214]	0.013 [-0.004; 0.030]	0.834	0.944	0.110
Fine motor	−1.611 [-3.515; 0.294]	0.017 [-0.012; 0.047]	0.720	0.870	0.150
Language	−1.889 [-3.630; −0.148]	0.021 [-0.006; 0.049]	0.707	0.905	0.198
Personal social behavior	−1.791 [-3.968; 0.385]	0.020 [-0.014; 0.054]	0.666	0.847	0.181

*CI*: confidence interval; *R* ^*2*^: coefficient of determination, indicating goodness of fit. *ΔR* ^*2*^: Represents the incremental coefficient of determination obtained by comparing the quadratic polynomial model with the linear model. *R*_*L*_^*2*^: Linear *R* ^2^; *R*_*Q*_^*2*^: Quadratic *R* ^2^.

The table reports coefficients and statistics for the linear (B_1_) and quadratic (B_2_) terms.

### Trio whole exome sequencing results

Trio whole exome sequencing (Trio-WES) identified a *de novo* heterozygous deletion in the proband (Table 4). The deletion spanned approximately 2.06 Mb, corresponding to seq [GRCh37] del (Denommé-Pichon et al., 2023) (p13.3p13.2)×1, chr17:g.1707883_3765621del. Notably, this 2.06 Mb deletion did not encompass the *YWHAE* or *CRK* genes, which is consistent with ILS and excludes a diagnosis of MDS. This variant was absent in the proband’s parents and elder brother. In accordance with the ClinGen/ACMG guidelines for copy number variant interpretation, the deletion was classified as pathogenic (P) based on evidence PVS1, PS2, PM2, and PP1 (Riggs et al., 2020).

**TABLE 4:** Results of WES in the proband, his parents and elder brother.

​	Proband	Brother	Mother	Father
Sex	Male	Male	Female	Male
Age of diagnosis	1y8m	1y8m	35y3m	38y5m
Variant position (reference GRCh37/hg37)	chr17:1707883–3765621	NA	NA	NA
Variation size	2.06 Mb	NA	NA	NA
Chromosomal location	17p13.3p13.2	NA	NA	NA
Variation pattern	del	NA	NA	NA
Origin	*De novo*	NA	NA	NA
*YWHAE* gene	Normal	Normal	Normal	Normal
*CRK* gene	Normal	Normal	Normal	Normal
*PAFAH1B1* gene	Haploinsufficiency	Normal	Normal	Normal
Diagnosis	ILS	NA	NA	NA

This CNV, was classified as pathogenic (P) according to 2020 ClinGen/ACMG, guidelines, supported by PVS1 (*PAFAH1B1* haploinsufficiency), PS2 (*de novo*), PM2 (absent in controls), and PP1 (consistent ILS, phenotype).

### CNV-seq validation findings in the proband

**FIGURE 4:**
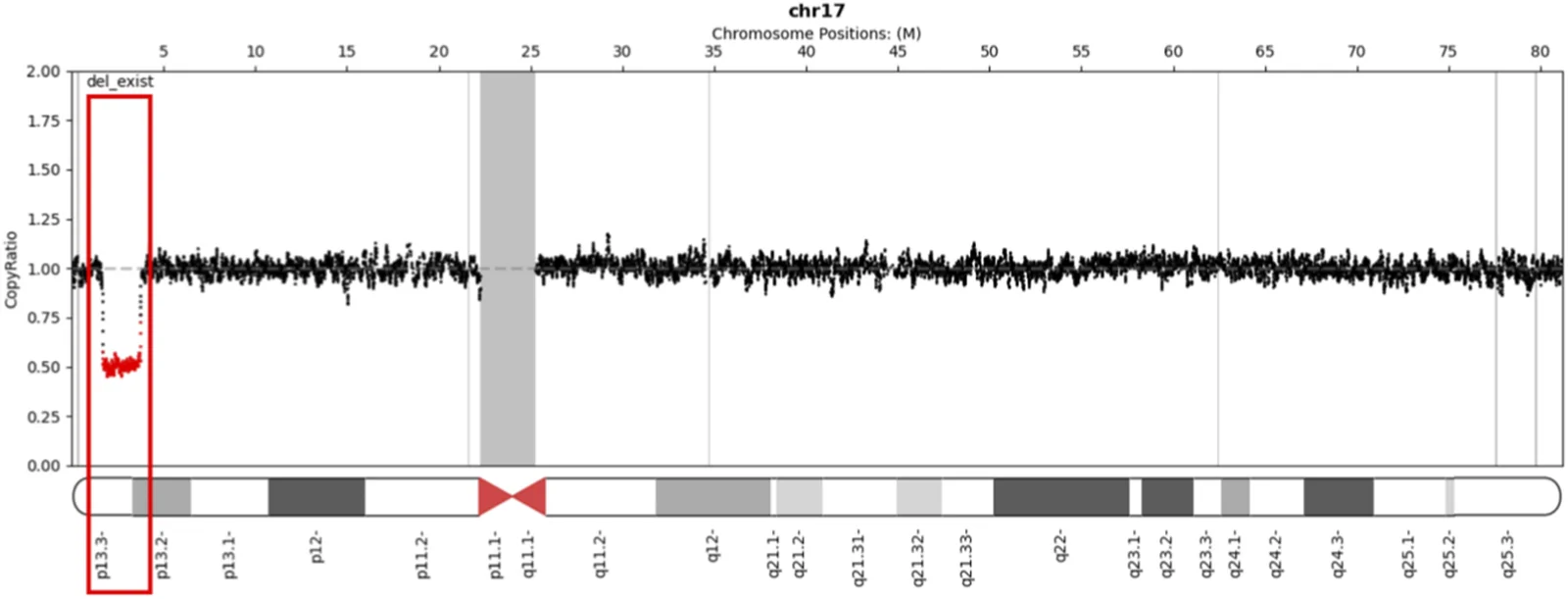
CNV-Seq validation of a single-copy deletion in the chromosome 17p13.3p13.2 region of the proband. CNV-Seq analysis identified a heterozygous deletion (indicated by the red box) in the proband, with the copy ratio dropping to approximately 0.5, consistent with a single-copy loss of the 17p13.3p13.2 region (chr17: g.1707883_3765621del, GRCh37). The horizontal axis indicated the physical position on chromosome 17 (in megabases, Mb), and the vertical axis represented the copy ratio.

Copy number variation sequencing (CNV-Seq) was subsequently performed to validate the WES findings, confirming a *de novo* heterozygous deletion in the 17p13.3p13.2 region of the proband (Figure 4). The copy ratio dropped to approximately 0.5, consistent with previous results.

### Genotype-phenotype correlation analysis of genes in the deleted region

To prioritize the pathogenic candidate genes most strongly associated with the ILS phenotype, the present study performed haploinsufficiency pathogenicity and functional constraint analyses on all protein-coding genes within the proband’s microdeletion region. These analyses were applied to evaluate gene dosage sensitivity and evolutionary constraint strength. The pHaplo score reflects the pathogenic risk of gene haploinsufficiency, with higher scores indicating a greater likelihood that haploinsufficiency is pathogenic. The pLI score quantifies the constraint intensity of genes against loss-of-function (LoF) variants, where a pLI ≥0.9 indicates high intolerance to LoF mutations.

**FIGURE 5:**
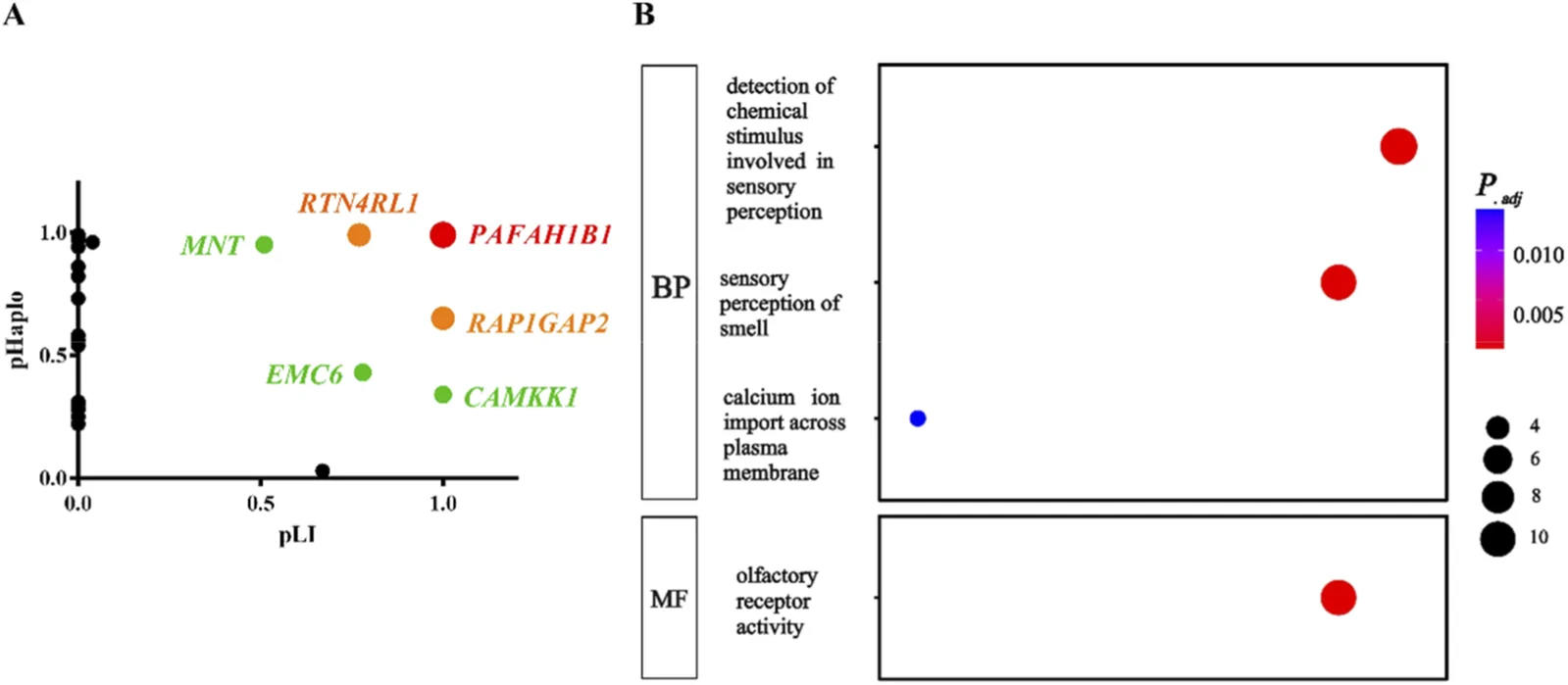
Pathogenicity assessment and functional enrichment analysis of genes in the 17p13.3p13.2 deletion region. **(A)** Scatter plot of haploinsufficiency scores (pHaplo) vs. probability of loss-of-function intolerance (pLI) for genes in the deletion region. Key pathogenic genes (*PAFAH1B1*(red), *RTN4RL1* (orange), *RAP1GAP2* (orange)) and secondary candidate genes (green, e.g., *MNT*, *EMC6*, *CAMKK1*) are labeled. **(B)** Bubble plot of GO functional enrichment analysis for genes in the deletion region. The y-axis shows GO terms grouped into Biological Process (BP) and Molecular Function (MF); the color of the bubbles represents the adjusted *P*-value (*P.*_*adj*_), and the size represents the number of enriched genes. Significantly enriched terms include pathways related to detection of chemical stimulus involved in sensory perception, calcium ion transmembrane transport, and olfactory receptor activity.

As shown in Figure 5A, most genes clustered in the low pHaplo and low pLI region, suggesting weak pathogenicity of haploinsufficiency and low functional constraint. In contrast, a small subset of genes exhibited both high pHaplo and high pLI, forming the core candidate gene set. Among these, *PAFAH1B1*, *RAP1GAP2*, and *RTN4RL1* demonstrated high pLI (>0.9) and high pHaplo (>0.8), indicating high sensitivity to haploinsufficiency and strong functional constraint. Genes such as *MNT*, *EMC6*, and *CAMKK1* were classified as the secondary candidate gene set. Notably, *PAFAH1B1* was identified as the top-priority candidate, which is highly consistent with the known core clinical phenotypes of ILS.

In addition, to explore the potential functions of genes within the 17p13.3p13.2 deletion region of the proband, this study performed Gene Ontology (GO) enrichment analysis of the deleted genes, with an adjusted *P* value (*P*._*adj*_) < 0.05 as the significance threshold. As shown in Figure 5B, a total of four GO terms were significantly enriched, including three biological process (BP) terms and one molecular function (MF) term. In the BP category, the genes were mainly enriched in three pathways. These terms include detection of chemical stimulus involved in sensory perception (GO:0050907, *P*._*adj*_ = 1.3e-06), sensory perception of smell (GO:0007608, *P*._*adj*_ = 4.9e-06) and calcium ion import across plasma membrane (GO:0098703, *P*._*adj*_ = 0.014). These findings suggested that dysregulation in chemosensory perception and calcium homeostasis regulation pathways may play a critical role in the pathogenesis of ILS. In the MF category, the significantly enriched term was olfactory receptor activity (GO:0004984), indicating that these genes may potentially contribute to olfactory regulation. More *in vitro* and *in vivo* functional experiments will be needed to validate the relevant mechanisms in the future.

## Discussion

Isolated lissencephaly sequence (ILS) is an isolated, non-syndromic neurodevelopmental disorder caused by abnormal neuronal migration during embryonic development at 9–13 weeks of gestation (Lo Nigro et al., 1997). Its clinical features include a smooth cerebral cortical surface, agyria or pachygyria, and an absence of the characteristic craniofacial dysmorphism and multisystem involvement seen in Miller Dieker syndrome (MDS) (Mahendran and Brown, 2025). Multiple studies have demonstrated that copy number variations or point mutations affecting *PAFAH1B1* are the primary cause of ILS (Cardoso et al., 2002; Saillour et al., 2009a; Cardoso et al., 2000; Vallee et al., 2001). However, due to significant clinical heterogeneity and great difficulty in early genetic accurate diagnosis, most studies focus on cross-sectional phenotypic analysis, and long-term follow-up data to support genotype-phenotype correlation are lacking. This study identified a Chinese pediatric patient with ILS caused by a *de novo* heterozygous deletion at 17p13.3p13.2, and conducted the first 6-year longitudinal follow-up. Dynamic phenotypic changes and corresponding genotype-phenotype correlations of the proband were systematically analyzed, verifying *PAFAH1B1* haploinsufficiency as the core pathogenic mechanism of ILS. The patient exhibited progressive growth restriction and sustained neurodevelopmental decline across all domains. Furthermore, this research found that besides *PAFAH1B1*, co-deletion of olfactory and calcium regulatory genes may exert synergistic effects and aggravate related phenotypes. These findings expand the phenotype and genetic landscape of neurodevelopmental disorders associated with 17p13.3 microdeletion. It also provides reference for the early diagnosis and precise treatment of related diseases.

The application of Trio-WES combined with CNV-Seq can precisely locate the size and variant loci of deleted fragments, significantly improving the diagnostic level of complex developmental diseases (Zhang et al., 2024; Zhai et al., 2021; Zeng et al., 2025). Compared with traditional single detection methods, the combined approach overcomes the limitations of individual methods, allows rapid identification of pathogenic etiologies, and significantly improves the diagnostic yield and accuracy of rare neurodevelopmental genetic disorders (Zhang et al., 2024; Hu et al., 2020; Jiao et al., 2019). In this study, the combined approach accurately identified the clinical features and variant in the proband. It is recommended that a standardized combined detection strategy be adopted for children with suspected hereditary neurodevelopmental disorders, such as global developmental delay, craniofacial malformations and other multiple phenotypes.

Clinically, ILS is a rare neurodevelopmental disorder characterized by lissencephaly. Its core clinical manifestations include developmental delay, myoclonic jerks, spasms and epilepsy (Chong et al., 1996; Leventer et al., 2001). Additional features include microcephaly, facial dysmorphism, growth retardation, muscle hypotonia, impaired motor coordination, dysphagia, respiratory difficulties, and intellectual disability (Chong et al., 1996; Leventer et al., 2001; Schinzel, 1988; Saillour et al., 2009b). Furthermore, brain MRI reveals moderate pachygyria, cerebellar hypoplasia, and associated agenesis of the corpus callosum (Kato and Dobyns, 2003; Leventer et al., 2001; Saillour et al., 2009b). In this study, the proband exhibited the core manifestations of ILS, including facial dysmorphism, microcephaly, plagiocephaly, severe developmental delay, muscle hypotonia, dysphagia, and intellectual disability, sensory abnormalities, cognitive impairment, feeding difficulties, among others. Based on a 6-year longitudinal follow-up, the findings observed that the proband’s height and weight increased gradually with age in accordance with the growth trend of healthy children. However, his height Z score decreased from −0.37 at 2 months to −1.92 at 57 months. Similarly, weight Z score decreased from −1.25 at 2 months to −1.79 at 57 months. This further supports impaired physical development. In addition, repeated Gesell developmental schedule assessments revealed that all neurodevelopmental domains showed an age-related reduction in DQ scores, and the decline slowed progressively over time. The adaptive behavior domain displayed the most marked alteration. This reflects the unique natural progression of ILS. The early rapid progressive neurodevelopmental delay is attributed to irreversible neuronal migration defects and cortical dysplasia caused by *PAFAH1B1* haploinsufficiency. The slowed declining rate in the later period indicates a stabilized disease state. Notably, the patient did not receive any rehabilitation training or intervention throughout the follow-up period. His clinical phenotype occurred in the absence of any support. Moreover, the proband presented with classic epileptic seizures this past May, and no seizure events had been documented previously. These findings suggest that ILS is not only clinically heterogeneous, but also that its phenotypic variability may be closely related to the specific genes involved. Through 6-year longitudinal follow-up, this study supplements the phenotype spectrum of children with ILS.

Genotype analysis confirmed that 17p13.3p13.2 *de novo* heterozygous deletion is a major cause of ILS in this proband (Ledbetter et al., 1992). In this study, the deletion region in the proband encompassed several important protein-coding genes, including *PAFAH1B1*, *RAP1GAP2*, *RTN4RL1, CLUH* and so on. Based on the genetic pHaplo and pLI scores, *PAFAH1B1* was identified as the top priority candidate gene. This suggested that haploinsufficiency of this gene is the main molecular basis underlying the core ILS phenotype in the proband (Mahendran and Brown, 2025; Bruno et al., 2010; Zhang et al., 2022). Previous studies have also demonstrated that copy number variations or point mutations affecting *PAFAH1B1* are the primary cause of ILS (Lo Nigro et al., 1997; Cardoso et al., 2002; Saillour et al., 2009a; Cardoso et al., 2000; Vallee et al., 2001). These mutations result in a relatively isolated phenotype which is consistent with the phenotypic characteristics of the proband. The *PAFAH1B1* gene is localized to the 17p13.3 region and encodes a highly conserved 410-amino-acid protein, designated as LIS1 or PAFAH1B1. It contains an N-terminal coiled-coil domain and seven C-terminal WD repeat sequences, both of which are involved in mediating protein-protein interactions (Leventer et al., 2001). Functional experiments have demonstrated that LIS1 binds to subunits of the dynein complex, thereby regulating its efficient assembly and targeting to microtubule ends, which consequently enhances dynein-mediated sliding (Singh et al., 2024; Splinter et al., 2012). This process is crucial for the proper migration of central neurons and the formation of layered cerebral structures (Nagamani et al., 2009). Notably, mouse models with heterozygous *PAFAH1B1*deletion also exhibit a series of phenotypes similar to those of human ILS, including defective neuronal migration, disrupted lamination of the cerebral cortex and hippocampus, impaired spatial learning, and epileptic seizures (Dinday et al., 2017). These findings provide direct functional evidence for the pathogenicity of *PAFAH1B1* haploinsufficiency. In addition, other highly scored genes, such as *RAP1GAP2*, *RTN4RL1*, and *CLUH*, may synergistically modify the clinical phenotype through dosage effects. Their specific mechanisms of action require further verification by functional experiments in the future.

To further explore the biological functions and potential pathogenic pathways of candidate genes, Gene Ontology (GO) enrichment analysis was conducted on protein-coding genes within the proband’s 17p13.3p13.2 deletion region. The results showed that candidate genes were significantly enriched in the detection of chemical stimulus involved in sensory perception, sensory perception of smell and calcium ion transmembrane transport pathways. This suggested that abnormalities in these pathways may play a key role in ILS. In this study, the pathway of detection of chemical stimulus involved in sensory perception is mainly mediated by olfactory perception-related genes, whose dysfunction is directly associated with olfactory impairment (DeMaria and Ngai, 2010). The 17p13.3p13.2 region contains multiple genes related to olfactory receptors (such as the *OR* family) (Malnic et al., 2004). ORs serve as the core chemoreceptors for olfactory perception. They also regulate gene expression, axon targeting, and neural circuit assembly in olfactory sensory neurons, playing dual roles in the development and function of the olfactory system (DeMaria and Ngai, 2010). Deletion of these genes directly causes functional defects in chemosensory receptors, which is highly consistent with the abnormal sensory phenotypes observed in the proband. In addition, calcium homeostasis is essential for neuronal migration, axon growth, and synaptic signal transmission (Leclerc et al., 2012; Díaz-Piña et al., 2024). The 17p13.3p13.2 region harbors multiple calcium channels and homeostasis-regulating genes, including *TRPV1*, *PAFAH1B1*, *P2RX5*, and *CAMKK1* (Kholmanskikh et al., 2006; Khakh et al., 2006; Tokumitsu and Sakagami, 2022; Haustrate et al., 2020). Deletion of these genes may disrupt calcium homeostasis, thereby impairing nervous system development and signal transduction, leading to deficits in cognitive function, learning, and memory (Berridge et al., 2003). Previous studies have confirmed that haploinsufficiency of *PAFAH1B1* is the primary cause of neuronal migration disorders and the lissencephaly phenotype (Mahendran and Brown, 2025; Bruno et al., 2010; Zhang et al., 2022). The present study indicates that, besides *PAFAH1B1*, co-deletion of olfactory and calcium regulatory genes may exert potential synergistic effects and may aggravate related phenotypes. Such phenotypes include sensory deficits, neurodevelopmental delay, and cognitive impairment, which in turn worsen pathological progression of ILS. For instance, the development and functional maintenance of olfactory sensory neurons are closely linked to calcium homeostasis (DeMaria and Ngai, 2010; Solís-Chagoyán et al., 2016). Co-deletion of olfactory genes (such as the OR family) and calcium regulatory genes may synergistically disrupt neuronal function via receptor perception and signal transduction (DeMaria and Ngai, 2010). However, the association between these gene deletions and aggravated phenotypes has not been confirmed, owing to the absence of rigorous *in vitro* and *in vivo* functional validation. Future research may focus on sensory perception-related chemical stimuli and calcium ion transmembrane transport in neurodevelopmental disorders. This will help systematically elucidate the molecular pathogenesis of 17p13.3p13.2 deletion related disorders and provide a theoretical basis for developing precise therapeutic targets.

### Limitations

Firstly, the number of patients included in this study was limited, as this was a single-center, single-case study. Further studies with expanded sample sizes and combined multicenter long-term follow-up data are required to better validate the phenotypic heterogeneity and genotype-phenotype correlations of ILS. Secondly, the proband’s height and weight records as well as the DQ assessments for different follow-up periods are only recorded once, which may result in certain statistical errors. Thirdly, this study did not perform *in vitro* functional experiments to verify the specific regulatory mechanisms in sensory perception and/or calcium ion transmembrane transport pathways. Basic research such as cellular assays and animal models is needed to further elucidate the underlying mechanisms in the future. Lastly, the present follow-up did not address adolescent neurodevelopment, or long-term prognosis of the patient. We will continue to monitor the patient’s clinical progression to provide evidence-based evidence for the intervention and treatment of ILS.

## Conclusion

Isolated lissencephaly sequence (ILS) associated with 17p13.3 microdeletion represents a severe congenital neurological disorder characterized by prominent neurodevelopmental deficits and impaired physical growth. In this 6-year longitudinal follow-up study, we demonstrated that the *de novo* 2.06 Mb heterozygous deletion at 17p13.3p13.2 was responsible for progressive growth restriction and continuous deterioration of neurodevelopment in all functional domains in the proband. Haploinsufficiency of the *PAFAH1B1* gene was confirmed as the primary pathogenic mechanism, whereas co-deletion of olfactory and calcium-regulatory genes may further contribute to phenotypic complexity and severity. Collectively, our findings broaden the phenotypic and genetic spectrum of ILS in the Chinese population, and offer important evidence for early molecular diagnosis, prognostic assessment, and personalized genetic counseling for affected patients and their families.

## Data Availability

The datasets generated and/or analysed during the current study are not publicly available for ethical reasons, but are available from the corresponding author on reasonable request.
